# Proximity to Goat Farms and *Coxiella burnetii* Seroprevalence among Pregnant Women

**DOI:** 10.3201/eid1712.110738

**Published:** 2011-12

**Authors:** Wim van der Hoek, Jamie C.E. Meekelenkamp, Frederika Dijkstra, Daan W. Notermans, Ben Bom, Piet Vellema, Ariene Rietveld, Yvonne T.H.P. van Duynhoven, Alexander C.A.P. Leenders

**Affiliations:** National Institute for Public Health and the Environment, Bilthoven, the Netherlands (W. van der Hoek, F. Dijkstra, D.W. Notermans, B. Bom, Y.T.H.P. van Duynhoven);; Jeroen Bosch Hospital, ’s-Hertogenbosch, the Netherlands (J.C.E. Meekelenkamp, A.C.A.P. Leenders);; Animal Health Service, Deventer, the Netherlands (P. Vellema);; Municipal Health Service “Hart voor Brabant,” ’s-Hertogenbosch (A. Rietveld)

**Keywords:** Q fever, Coxiella burnetii, seroprevalence, pregnancy, goats, the Netherlands, bacteria, vector-borne infections

## Abstract

During 2007–2009, we tested serum samples from 2,004 pregnant women living in an area of high Q fever incidence in the Netherlands. Results confirmed that presence of antibodies against *Coxiella burnetii* is related to proximity to infected dairy goat farms. Pregnant women and patients with certain cardiovascular conditions should avoid these farms.

Dairy goat farms were implicated in the large Q fever epidemic (>3,500 human cases) in the Netherlands during 2007–2009 (*1**,**2*). However, most human infections remain asymptomatic or appear as a self-limiting febrile illness and are therefore not reported. Seroprevalence studies are needed to discover the true infection pressure in the population. We aimed to establish whether the presence of antibodies to *Coxiella burnetii*, the etiologic agent of Q fever, is associated with physical proximity to infected small ruminant (dairy sheep and goat) farms.

## The Study

Serum samples from pregnant women were obtained from laboratories located in the high-incidence Q fever area in the province of Noord-Brabant. The samples had been collected during June 20, 2007–May 26, 2009, during a screening program for syphilis, hepatitis B, and HIV infection, which was routinely offered to all pregnant women. Serum samples were analyzed by using an immunofluorescence assay (Focus Diagnostics, Cypress, CA, USA) for detecting IgG and IgM against phases I and II of *C. burnetii* infection in a single dilution of 1:64. An IgG II titer >64 was considered indicative of past infection. Possible recent infection was defined as IgM II >64 combined with either an IgG II or IgM I titer >64.

**Figure 1:**
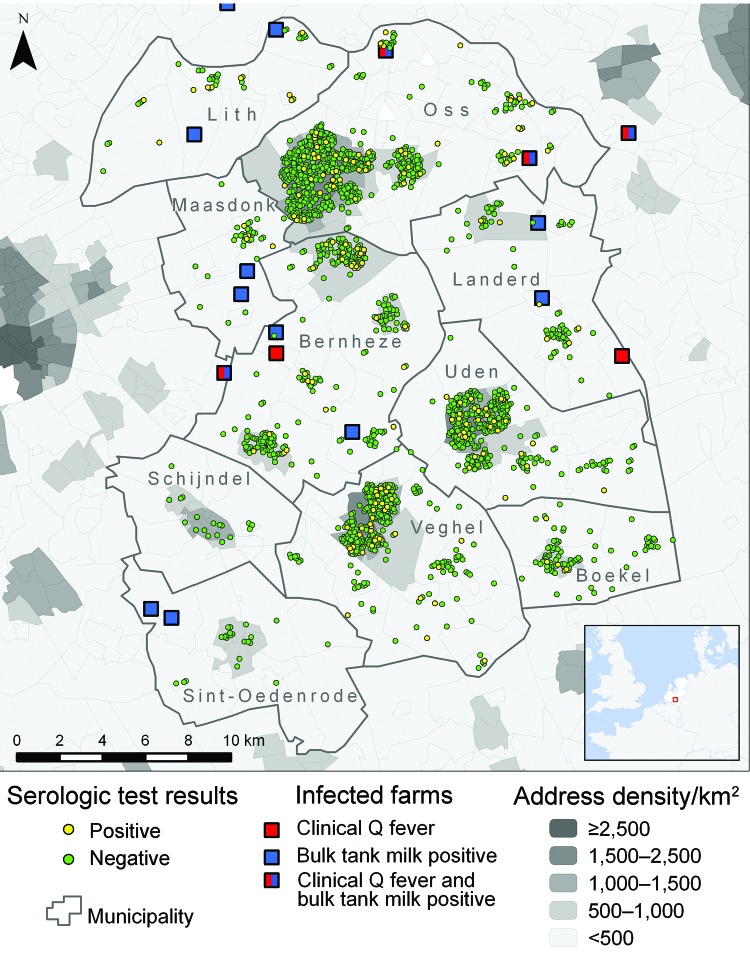
Location of the 10 municipalities studied in southern area of the Netherlands, 2007–2009, with residence and serologic results for 2,004 pregnant women, sites of small ruminant farms with infected animals, and address density. Three of the 20 farms included in the analysis are not visible.

**Figure 2:**
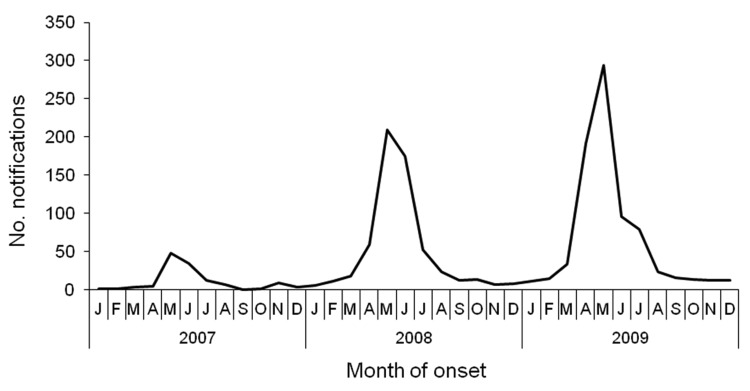
Q fever notifications by month of onset of illness in 10 municipalities in southern area of the Netherlands, 2007–2009

We included 10 adjacent municipalities (Figure 1) that had an incidence of Q fever notifications over the study period of 0.5–19.6 per 1,000 population and with clear seasonal peaks (Figure 2). Median age of the 2,004 women in the study was 30 years (interquartile range 27–33 years) with no significant differences between the municipalities. Seroprevalence for past infection was 9.0% (181/2,004), ranging from 0% to 21% between the 10 municipalities (Table 1). In 57 (31%) of 181 women, IgM II titer was >64. Only 2 women had an IgM II titer >64 and IgM I titer >64 without an IgG II titer >64. Seroprevalence for possible recent infection was therefore 2.9% (59/2,004), with a range of 0%–10% between the 10 municipalities.

**Table 1:** Antibodies to *Coxiella burnetii* in pregnant women in 10 municipalities in Noord-Brabant Province, the Netherlands, June 2007–May 2009

Municipality	Total population	No. pregnant women tested	Serologic profile, no. (%) women	
			IgG II titer >64	Possible recent infection*
Lith	6,667	42	9 (21.4)	4 (9.5)
Oss	77,097	702	57 (8.1)	22 (3.1)
Maasdonk	11,260	38	5 (13.2)	0
Bernheze	29,615	291	30 (10.3)	15 (5.2)
Landerd	14,805	72	6 (8.3)	4 (5.6)
Uden	40,360	380	32 (8.4)	8 (2.1)
Veghel	37,125	355	37 (10.4)	6 (1.7)
Boekel	9,692	84	5 (6.0)	0
Schijndel	22,889	18	0	0
Sint-Oedenrode	17,427	22	0	0
Total	266,937	2,004	181 (9.0)	59 (2.9)

*IgM titer to phase II antigen >64 combined with either an IgG II or IgM I titer >64.

For each pregnant woman, we calculated the distance from her home address to the closest farms in the following 3 categories: 1) dairy goat or dairy sheep farm where clinical Q fever (i.e., abortion waves) occurred during 2005–2009 (data from Animal Health Service) (8 farms [all goat farms] were identified in this way, of which 6 were located within or just outside of the study area) (Figure 1); 2) dairy goat or dairy sheep farms at which bulk tank milk tested positive for *C. burnetii* antibodies in the mandatory bulk tank milk monitoring program in 2009 (data from Food and Consumer Product Safety Authority; 12 goat farms were identified); and 3) a farm with >100 goats or sheep, irrespective of the infection status and production type (milk or meat) of the farm (data from the Ministry of Agriculture, Nature and Food Quality). Details of category 1 and category 2 farms are provided in Table A1.

Univariate regression analysis showed that pregnant women living <2 km from a farm that had experienced clinical Q fever had a higher risk of testing positive for antibodies to *C. burnetii* than those living >5 km away (odds ratio 2.63, 95% confidence interval 1.33–5.20 for IgG II titer >64 and odds ratio 6.58, 95% confidence interval 2.78–15.55 for possible recent infection). The increased risk for farms that had positive test results during monitoring of bulk tank milk was not significant. No increased risk was found for women who lived close to any farm with >100 animals. However, 98% of the population in the study area live within 5 km of such farms. In multivariate logistic regression analyses, taking into account address density of the neighborhood and other relevant variables, living <2 km from a farm with clinical Q fever remained a strong risk factor (Table 2).

**Table 2:** Multivariate logistic regression models for *Coxiella burnetii* IgG II seropositivity and serologic indication for possible recent infection based on house location of 2,004 pregnant women, the Netherlands, 2007–2009*

Variable	OR (95% CI)	
	IgG II titer >64	Possible recent infection†
Distance to nearest farm with clinical Q fever, km		
<2.0	2.38 (1.19–4.73)	6.68 (2.53–17.64)
2.0–4.9	1.12 (0.74–1.71)	2.82 (1.34–5.92)
>5	Reference	Reference
No. infected farms within 5 km		
>1		0.92 (0.46–1.85)
0		Reference
Total no. locations with sheep or goats within 5 km		
>140	1.29 (0.95–1.76)	
<140	Reference	
Address density of neighborhood, addresses/km^2^‡		
<500	1.85 (1.23–2.78)	1.32 (0.65–2.70)
>500	Reference	Reference

*OR, odds ratio; CI, confidence interval. Blank cells indicate variable not included. †IgM antibody titer to phase II antigen >64 combined with either an IgG II or IgM I titer >64. ‡Neighborhoods were categorized as not urban if the address density was <500 addresses/km^2^ and urban if >500/km^2^.

## Conclusions

The presence of antibodies against *C. burnetii*, especially levels suggesting recent infection, is associated with living near a farm with infected dairy goats. This finding applied only to farms where animals had clinical Q fever and not for farms where tests of bulk tank milk were positive. A study in 2008 showed that persons who lived near (<2 km) an infected dairy goat farm had a much higher risk for Q fever than did persons who lived further away (>5 km) (*3*). However, these results were based on notified cases, i.e., patients with clinical signs. Because the link to goat farming has received substantial attention in the public media, persons living close to goat farms may have sought medical care for suspected Q fever more rapidly than those who lived distantly from goat farms. Our population-based serologic study had a control group of seronegative women and identified asymptomatic infections. Combined, both studies provide evidence that living near dairy goat farms that experience abortion waves increases the risk in humans for symptomatic and asymptomatic *C. burnetii* infection.

A limitation of this study is that the exact infectious periods for each of the 20 farms in the study are unknown. Mandatory systematic monitoring of bulk tank milk only started in October 2009. However, for 17 of the 20 farms identified in the distance calculations in the present study, bulk tank milk testing results from 2008 were available from the records of the Animal Health Service. When an ELISA was performed, animals at 13 of the 17 farms tested positive for *C. burnetii* antibodies (Table A1). Furthermore, for 15 of the 17 farms, bulk tank milk tested positive by PCR in 2008. The assumption that persons became infected where they lived, although infection might have occurred elsewhere, might have weakened the association between house location and infected farms because of nondifferential misclassification. We did not account for circumstances that may play a role in transmission from farm to humans, such as wind, vegetation patterns, and soil conditions around infected farms (*4*). We assume that the effect of the voluntary vaccination program of small ruminants in 2008 had only limited effects on the study. Culling of pregnant animals on infected farms did not affect the results of the study because that began in December 2009.

We found an overall prevalence of IgG II antibodies of 9.0%. In comparison, during 2006–2007, a seroprevalence of 2.4% was found in a nationwide seroprevalence survey in the Netherlands, just before the first major outbreak (*5*). The present Q fever epidemic peaked right after the last data were collected for the present study. In the second half of 2009, seroprevalence for blood donors in the high-incidence area was estimated at 12.2% (*6*). The findings of the different seroprevalence studies are consistent with the view that Q fever newly emerged in the Netherlands, peaking in 2009, and that a high infection pressure has resulted in increased seroprevalence in the general population, including in pregnant women.

Whether infection during pregnancy is associated with adverse pregnancy outcomes remains uncertain. The international literature suggests this conclusion, but an analysis based on 1,174 of the 2,004 women included in the present study showed no evidence of adverse pregnancy outcome among women with antibodies to *C. burnetii* (*7**–**9*). Despite uncertainties surrounding the clinical significance of asymptomatic seropositivity, this study supports the recommendation that pregnant women and persons at risk for chronic Q fever, such as patients with certain cardiovascular conditions, should avoid visiting infected farms.

## Appendix

**Table A1:** Details of Q fever–affected dairy goat farms included in analysis of *Coxiella burnetii* seropositivity among pregnant woman, the Netherlands, 2007–2009*

Farm no.	No. goats in herd†	Date clinical Q fever detected	Month bulk tank milk found positive, 2009	PCR result, 2008	ELISA result, 2008
1	1,700	2006 Mar	None	NA	NA
2	604	2006 Apr	Nov	+	+
3	830	2006 Aug	None	+	+
4	1,484	2007 Apr	None	+	+
5	2,228	2007 Apr	Nov	+	+
6	756	2008 Apr	Nov	+	+
7	2,194	2008 Jul	Dec	+	+
8	1,213	2009 Sep	Nov	+	–
9	905	None	Nov	+	+
10	652	None	Nov	+	+
11	1,013	None	Nov	+	+
12	432	None	Nov	NA	NA
13	1,065	None	Nov	–	–
14	606	None	Nov	+	–
15	713	None	Nov	+	+
16	1,271	None	Nov	+	+
17	727	None	Nov	+	+
18	2,653	None	Nov	–	–
19	2,125	None	Nov	NA	NA
20	1,182	None	Dec	+	+

*NA, not available; +, positive; –, negative. †November 2008 count of the Ministry of Agriculture, Nature and Food Quality.
